# Synthesis and Characterizations of Melamine-Based Epoxy Resins

**DOI:** 10.3390/ijms140918200

**Published:** 2013-09-05

**Authors:** Laura Ricciotti, Giuseppina Roviello, Oreste Tarallo, Fabio Borbone, Claudio Ferone, Francesco Colangelo, Michelina Catauro, Raffaele Cioffi

**Affiliations:** 1 Dipartimento di Ingegneria, Università di Napoli ‘Parthenope’, INSTM Research Group Napoli Parthenope, Centro Direzionale Napoli, Isola C4, Napoli 80143, Italy; E-Mails: giuseppina.roviello@uniparthenope.it (G.R.); claudio.ferone@uniparthenope.it (C.F.); francesco.colangelo@uniparthenope.it (F.C.); raffaele.cioffi@uniparthenope.it (R.C.); 2 Dipartimento di Scienze Chimiche, Università degli Studi di Napoli “Federico II”, Complesso Universitario di Monte S. Angelo, via Cintia, Napoli 80126, Italy; E-Mails: oreste.tarallo@unina.it (O.T.); fabio.borbone@unina.it (F.B.); 3 Dipartimento di Ingegneria Industriale e dell’Informazione, Seconda Università di Napoli, via Roma 29, Aversa 81031, Italy; E-Mail: michelina.catauro@unina2.it

**Keywords:** epoxy resin, melamine, glycidyl derivative, IR, DSC, DMA, glass transition, crosslinking reaction, synthesis

## Abstract

A new, easy and cost-effective synthetic procedure for the preparation of thermosetting melamine-based epoxy resins is reported. By this innovative synthetic method, different kinds of resins can be obtained just by mixing the reagents in the presence of a catalyst without solvent and with mild curing conditions. Two types of resins were synthesized using melamine and a glycidyl derivative (resins I) or by adding a silane derivative (resin II). The resins were characterized by means of chemical-physical and thermal techniques. Experimental results show that all the prepared resins have a good thermal stability, but differ for their mechanical properties: resin I exhibits remarkable stiffness with a storage modulus value up to 830 MPa at room temperature, while lower storage moduli were found for resin II, indicating that the presence of silane groups could enhance the flexibility of these materials. The resins show a pot life higher than 30 min, which makes these resins good candidates for practical applications. The functionalization with silane terminations can be exploited in the formulation of hybrid organic-inorganic composite materials.

## 1. Introduction

Epoxy resins are thermoset polymeric materials with a high crosslinking degree and that contain many hydroxyl groups [1]. Thanks to their unique combination of properties, such as good chemical and moisture resistance, low thermal shrinkage and high dielectric properties, epoxy resins are widely employed in modern industries, as adhesives, coatings and paints, substrates for printed circuit boards and encapsulating materials for microelectronic devices. Moreover, these thermosetting materials are used for the production of composite materials in which they usually act as the matrix, since they exhibit excellent adhesion to reinforcement, cure with low shrinkage, provide good dimensional stability and possess good mechanical properties [2–6]. In this respect, it is worth pointing out that very recently, some papers [7–10] and patents [11] have reported on the use of these resins to realize geopolymer-based hybrid composites (geopolymers are a class of synthetic inorganic aluminosilicate materials generally formed by reaction of an aluminosilicate with a silicate solution under strong alkaline conditions) for the production of low-cost and lightweight thermo-insulating panels or for their application in the field of restoration and masonry repair.

Epoxy-based resins can be obtained by means of a wide variety of chemical reactions and chemical reagents. They are commonly classified in five chemical classes: glycidyl ether, glycidyl esters, glycidyl amines, linear aliphatic- and cycloaliphatic-based resins [12–16]. For example, the reaction between bisphenol A and epichlorohydrin produces a widely used resin characterized by useful technological properties, such as good toughness and remarkable resistance to chemical and physical agents.

Another class of widely used thermosetting resins-based materials are obtained from the reaction of formaldehyde with melamine (1,3,5-triazine-2,4,6-triamine) [17]. These melamine-based resins have found several applications, since melamine allows the obtaining of polymers with relatively high thermal stability and good dielectric properties [18–22]. Melamine resins are used, for example, in the production of kitchen tools and plates, for coatings and floor covering.

The main disadvantage of melamine is its poor solubility in water and in most organic solvents, which makes it difficult to use directly [23]. In fact, the synthetic procedure commonly used in the polymer industry in the production of melamine-formaldehyde resins consists in the “activation” of the melamine ring by formation of a melamine-formaldehyde derivative. This is usually used as a precursor to synthesize resins with different functional groups or to allow the reaction to keep going, leading to a crosslinked resin [24].

Alternatively, some recent papers report on the production of melamine-based materials by means of a “reactive solvent”, such as formaldehyde and cyclohexanone, which can both dissolve and react with melamine, promoting the reaction [25,26]. Other papers report on the investigation of catalytic conditions able to promote the polymerization reaction [27–29]. In addition, melamine can be acetylated when heated in the presence of acetic anhydride [30]. Furthermore, in this case, the acetylated compound can be used as a building block for further functionalization and crosslinking reactions.

In this respect, it is worth noting that despite the great technological and industrial interest in melamine-based resins, only a few reports on epoxy resins based on melamine obtained by reaction between melamine and glycidyl derivatives are given in the literature.

Recently, oligo- and poly-etherols have been formed using melamine and oxirane in excess. This reaction can be carried out through two principal synthetic routes both, requiring complex and expensive procedures: the first one involves the presence of a high-boiling solvent, generally dimethylsulfoxide (DMSO), which must be further removed from products. This removal is usually carried out by reduced pressure distillation, due to the formation of decomposition byproducts in the reaction conditions [31]. The second one needs to be carried out at 120–130 °C, and a careful temperature control is needed, due to a strong exothermic effect that leads to a spontaneous increasing of the temperature up to 200–230 °C [31].

For these reasons, it seemed interesting to develop a new synthetic strategy for the production of melamine-based epoxy resins characterized by an easy and cost-effective procedure. In particular, in the present paper, we report the synthesis of two different types of melamine-based epoxy resins: the first one obtained by reacting melamine with a glycidyl derivative; the second one by adding also a silane derivative. This has been introduced to improve the compatibility of the resin with Si-based matrices for the development of organic-inorganic composite materials. According to the innovative synthetic strategy we propose, melamine resins can be prepared just by mixing the reagents in the presence of a catalyst, without solvent, and cured in mild conditions. Moreover, although thermally activated, all the reactions involved in the process do not develop uncontrolled exothermic effects, thus avoiding any overheating of the reaction mixture that would require a further thermal control.

Finally, all the prepared materials were characterized for their chemical-physical and mechanical properties.

## 2. Results and Discussion

### 2.1. Syntheses

Two resin types (indicated as resin (I) and resin (II)) with a different molar ratio of the reagents were investigated in order to evaluate the influence of the different chemical compositions on their reticulation, chemical-physical and mechanical properties. Thus, for both the resins, two preparations were set up, differing the stoichiometric ratio of the reagents. In particular, for the resin (I-a), the molar ratio between *N*,*N*-diglycidyl-4-glycidyloxyaniline and melamine was 1:2.7, while for the resin (I-b), 1:0.9. In the same way, the resin (II-a) was prepared by using *N*,*N*-diglycidyl-4-glycidyloxyaniline, melamine and (3-aminopropyl)trimethoxysilane (APTES) in the molar ratio of 1:2.2:9.3, while in the case of the resin (II-b), this ratio was 1:0.7:0.5.

**Scheme 1:**
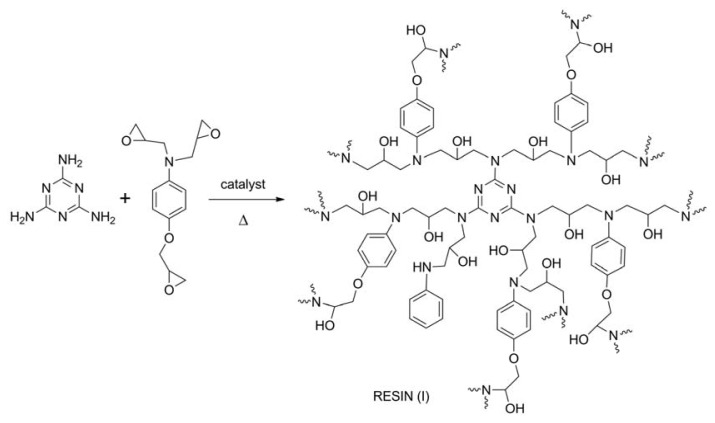
Synthesis reaction for the resin (I).

The resin (I) (see Scheme 1) was prepared by mixing the glycidyl compound and melamine at 60 °C in order to facilitate the consecutive addition and dissolution of 4-pyrrolidinopyridine in catalytic amount. In fact, solvent use was completely avoided in this procedure, and the catalyst dissolution in the reaction mixture was promoted by the temperature increase. After adding the catalyst and keeping the mixture at 60 °C for about 10 min, the system became more viscous and workable, and its color switched from white to light yellow. The “pre-crosslinked” system remains workable for at least 30 min (pot life) and can be shaped in the required form and spread on different kinds of surfaces.

Finally, in order to conclude the crosslinking process, the temperature was increased to 80 °C and kept for 3 h. After this thermal treatment, the resin assumed a yellow-orange color and became hard, tough and no longer workable.

**Scheme 2:**
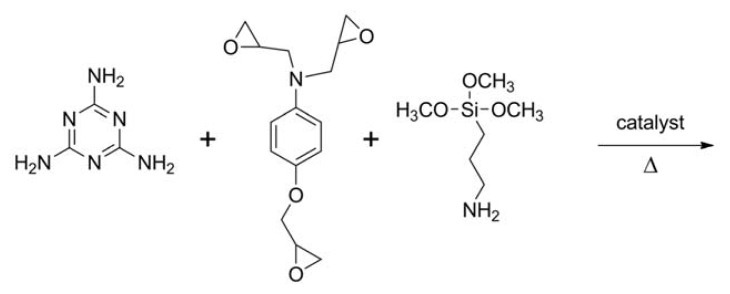

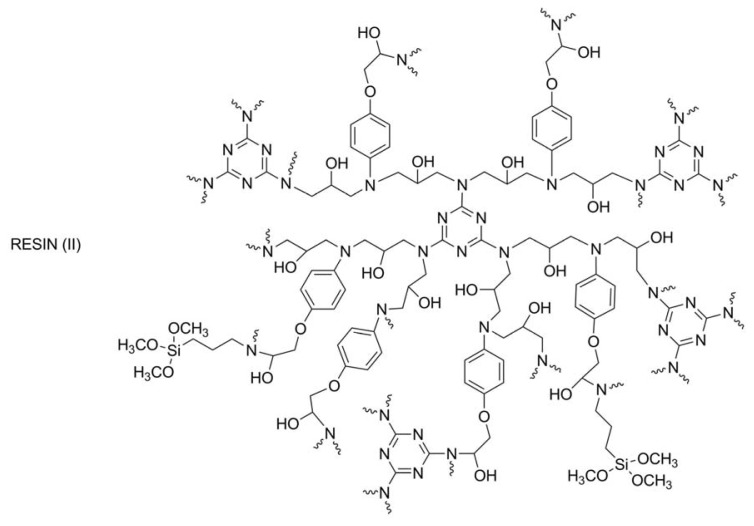
Synthesis reaction for the resin (II).

In a similar way, the resin (II) (see Scheme 2) was prepared first by mixing melamine and a glycidyl derivative at room temperature and then adding APTES and a catalytic amount of NaOH. At this point, the temperature was increased up to 60 °C and kept for 20 min to facilitate the reagent’s dissolution, since the reaction process was still solvent-free. As described above, the procedure requires a thermal treatment at 60 °C to improve the viscosity and workability of the system, and also, in this case, the pot life was about 30 min. At the end, the temperature was increased to 80 °C, and after 20–25 min, the resin became white, hard, tough and no longer workable.

### 2.2. Characterizations

#### 2.2.1. Thermogravimetric Analysis (TGA)

**Figure 1:**
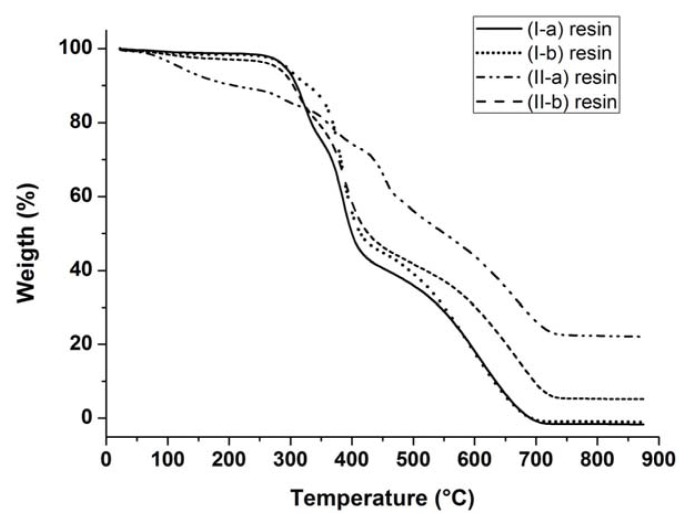
Thermogravimetric analyses (TGA) curves of the obtained resins.

Thermogravimetric analyses were performed on the obtained resins to compare their thermal stability (Figure 1).

It is observed in Figure 1 that the resins, (I-a), (I-b) and (II-b), show a degradation mechanism involving three main steps, while resin (II-a) shows a more complex degradation mechanism. The resin (I-a) is thermally stable up to about 240 °C. Above this temperature, a first degradation step can be observed until ≈340 °C, resulting in a weight loss of 24%. The second degradation process is completed at about 420 °C, with a weight loss of 56%. The final degradation step is completed at about 740 °C, and no combustion residue remains. In a similar way, resins (I-b) and (II-b) remain thermally stable up to about 250 and 240 °C, respectively. Furthermore, in these cases, the first degradation step is completed at ≈360°C, resulting in a weight loss for resins (I-b) and (II-b) of 15% and 23%, respectively. The second degradation process ends at ≈450 °C, with a weight loss of 55%. The final degradation step for the resin (I-b) is completed at about 720 °C, and no combustion residue remains, while for (II-b), the same step finishes at about 745 °C, with a combustion residue of 6%, due to silicon presence in the form of oxide. Unlike the other resins, the (II-a) one shows a more complex degradation mechanism in which a continuous mass loss is observed during heating. In particular, the first degradation step that finishes at ≈180 °C is present, corresponding to a weight loss of about 10%. This behavior is likely to be ascribed to the removal of methanol from the system as a result of an endothermic *trans*-alkoxylation reaction between the Si-OCH_3_ groups of silane and OH units present in the resin [32]. This hypothesis is supported also by IR and DSC analyses, which will be detailed in the following paragraphs. A similar behavior can also be seen for the resin (II-b), but the phenomenon is less pronounced, due to the lower amount of silane in the resin. Finally, for the resin (II-a), a 50% mass loss takes place at 550 °C, and the degradation mechanism is completed at about 790 °C. Moreover, as has already been seen in the case of the resin (II-b), a combustion residue of 22%, due to silicon presence in the form of oxide, was found.

Degradation temperatures and weight losses for all the studied systems are summarized in Table 1.

#### 2.2.2. Differential Scanning Calorimetry (DSC)

**Figure 2:**
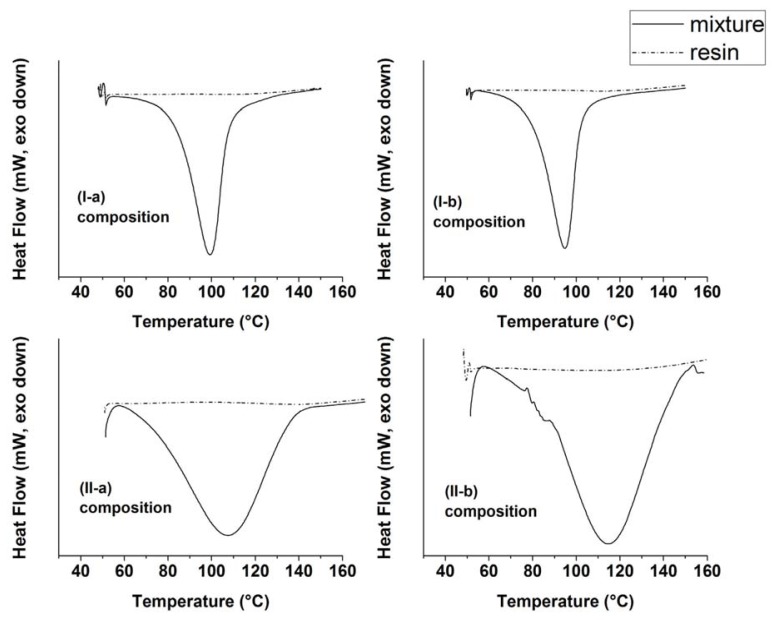
Representative differential scanning calorimetry (DSC) thermographs of the reaction mixtures before (continuous lines) and after (dotted lines) thermal treatment at 80 °C for three hours.

The thermal behavior of the epoxy melamine resins was examined by means of DSC analyses. Figure 2 shows DSC thermographs of the resins before and after the curing process. The point of maximum slope in the exothermic curve of the uncured epoxy resin system was chosen as the curing temperature (observed to be 80 °C). As evident from the shape of the exothermic peak in the DSC curves, the crosslinking process can be considered complete at about 150 °C, as observed in the scans for all the resins. Concerning cured samples, no residual heat of curing can be detected in the diagram of cured resins, hence indicating that the curing was completed in the selected conditions (80 °C, 3 h). By comparing the exothermic behavior of the uncured resins, it can be seen that the exothermic peak temperatures of resins (I-a) and (I-b) (see Table 2) are lower than those of (II-a) and (II-b), and the corresponding curing peaks appear more sharp, with a higher heat of reaction, ΔH. These values are listed in Table 2. This difference in the heat of reaction values could be due to a partial loss of methanol, as pointed out also by thermogravimetric and FT-IR (see next section) characterizations, with a further crosslinking reaction between silicon atoms and OH groups. In fact, lower ΔH values for (II-a) and (II-b) compositions could be in agreement with a endothermic *trans*-alkoxylation reaction that takes place during the heating process and subtracts heat to the whole reticulation process.

#### 2.2.3. FT-IR Analysis

**Figure 3:**
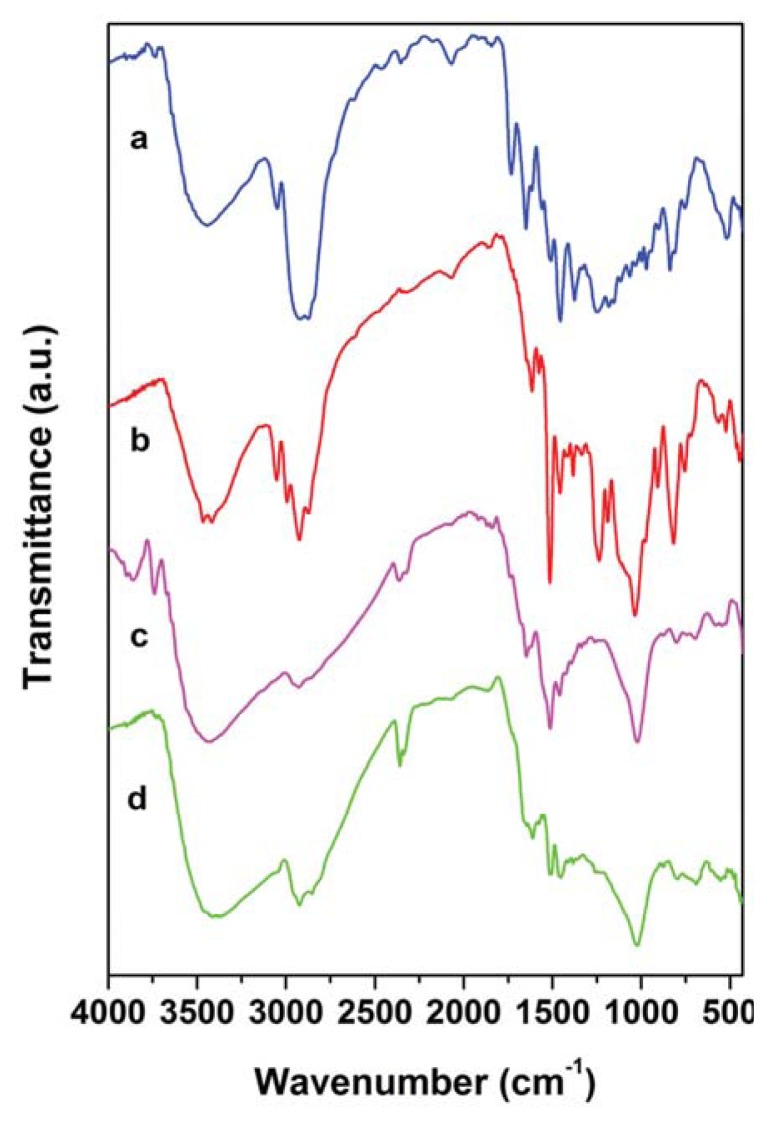
FT-IR spectra of the epoxy melamine resins: (**a**) Resins (I-a); (**b**) Resin (II-b); (**c**) Resin (II-a) and (**d**) post-cured resin (II-a) at 180 °C for 2 h.

The FT-IR spectra of the melamine resins are shown in Figure 3.

For all the spectra, the presence of the broad band at ≈3400 cm^−1^ is assigned to O-H stretching of the hydroxyl group [31,33]. The signals in the wavenumber range, 3000–2798 cm^−1^, are due to –CH_2_– symmetric and asymmetric stretching [31,33]. Moreover, it is worth pointing out that the NH bands at 3300 and 3130 cm^−1^ (for primary amine), 3288 and 733 cm^−1^ (for secondary amine) of starting melamine are absent, indicating that the all the amino-groups have reacted. In addition, the absence of bands at 971, 917 and 775 cm^−1^, due to terminal epoxy rings, reveals a satisfactory degree of crosslinking of the resins [34,35]. As expected, the bands of C=N belonging to 1,3,5-triazine rings at 1640–1450 cm^−1^ can be found in all the spectra. In addition, for resin (I-a), some weak bands, due to the aliphatic chain in the wavenumber range, 1250–1150 cm^−1^, can be observed. The presence of primary and secondary hydroxyl groups is demonstrated by their bands in the range, 1065–972 cm^−1^ [30].

Regarding resins (II-a) and (II-b) in which there is the presence of a silane derivative, an additional band is located at 1036 cm^−1^, due to the stretching vibration of the Si-O aliphatic groups. The low intensity band at 3740 cm^−1^ in the (II-a) spectrum could be likely ascribed to isolated Si-OH groups in the resin, as a result of a partial hydrolysis of Si-OCH_3_ units. After a thermal treatment of the sample at 180 °C for 2 h, this band disappeared (see d curve in Figure 3), and that located at 1036 cm^−1^ slightly broadened. As already described in the previous sections, this behavior could be caused by further crosslinking reaction promoted by the post-curing treatment, which may favor the later formation of the Si-OR groups through a *trans*-alkoxylation process.

#### 2.2.4. Dynamic Mechanical Analysis (DMA)

**Figure 4:**
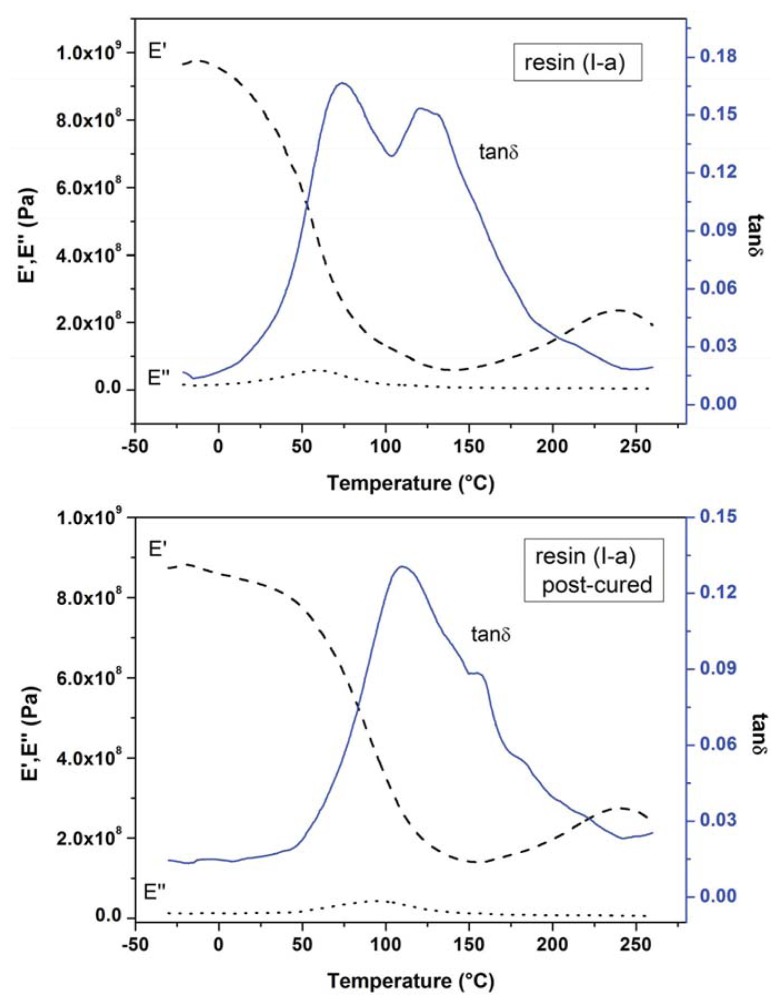

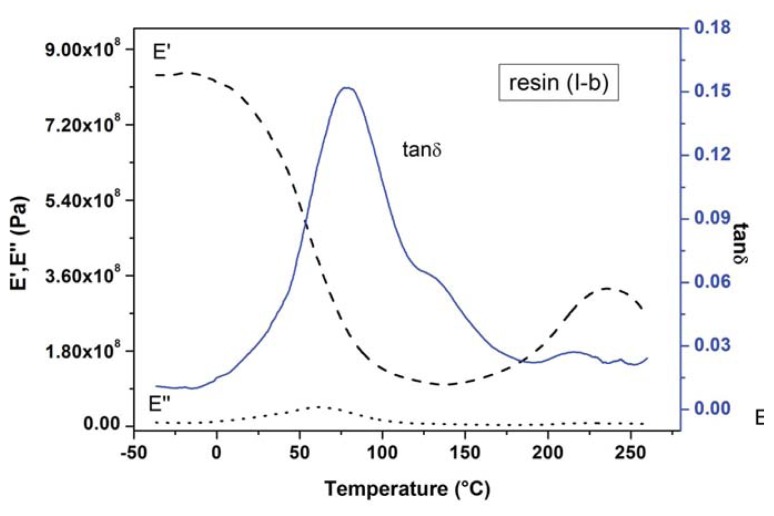
Dynamic mechanical analyses (DMA) curves for the neat resin (I-a), post-cured resin (I-a) at 120 °C for 2 h and resin (I-b). Solid blue line: tan δ; dashed line: E′ (storage modulus); dotted line: E″ (loss modulus).

The dynamic mechanical properties of the epoxy melamine resins were examined by DMA in the −30–260 °C temperature range. Figure 4 shows the storage modulus, E′, loss modulus, E″ and tan δ as a function of temperature for the neat resin (I-a), post-cured sample at 120 °C for 2 h and resin (I-b).

Figure 4 shows a broad peak with two maxima at 74 and 126 °C for tan δ of resin (I-a). The first one corresponds to the loss modulus peak at 58 °C and can be attributed to the glass transition temperature (*T*_g_) of the resin. Moreover, the second peak could be due to completion of the reticulation process, which occurs during the thermal treatment. In order to confirm this hypothesis, DMA analysis for the post-cured sample was performed. It is shown also in Figure 4 that this sample exhibits a different profile: there is one main peak at higher temperatures with just slight side shoulders. Thus, the resin after the thermal treatment shows a more extended curing degree and a higher glass transition temperature (*T*_g_ = 110 °C). For the resin (I-b) (see Figure 4), tan δ shows instead just one peak with one small side shoulder at a higher temperature (*T*_g_ = 79 °C), indicating that an advanced degree of crosslinking is already reached, and no further curing is needed. Storage modulus at room temperature and 150 °C, loss modulus peaks and glass transition temperatures for all the studied systems are summarized in Table 3. Since the storage modulus reflects the stiffness, the resins, (I-a) and (I-b), exhibit a comparable flexibility. Based on these observations, it can be affirmed that the molar ratio of 1:0.9 between the glycidyl derivative and melamine in the resin (I-b) allows one to obtain a melamine-based epoxy resin with an advanced curing degree and with mechanical and thermal properties comparable to that of resin (I-a) (in which the glycidyl derivative is in excess) without post-curing treatment.

DMA analysis carried out on resins (II-a) and (II-b) showed the same behavior as resins I; thus, glass transition temperatures were derived from post-cured samples at 100 °C for 2 h (see Table 3). Finally, whereas the resin (II-b) exhibits storage modulus values similar to those of resins I (672 MPa and 158 MPa at 25 °C and 150 °C, respectively), the lowest values of 137 and 67 MPa were observed for resin II-a.

Furthermore, in this case, the different thermal and mechanical properties of the resins could be related to their chemical composition: the excess of APTES, in resin (II-a) (in which the molar ratio between the glycidyl derivative, melamine and silane is 1:2.2:9.3, while in the resin (II-b), it is 1:0.7:0.5) leads to the obtainment of a material with lower stiffness, probably due to the presence of a higher amount of flexible silane tails.

## 3. Experimental Section

### 3.1. Materials and Methods

All reagents were purchased from Sigma Aldrich and used without further purification. Thermogravimetric analyses (TGA) were performed by a TA Instrument SDT2960. The thermographs were obtained with a heating rate of 10 °C/min using ≈10 mg of the powdered sample under air flow. Differential scanning calorimetry (DSC) measurements were performed by a Perkin Elmer Pyris 1 under dry nitrogen flow with a temperature-scanning rate of 5 °C/min. FT-IR measurements were performed using a Jasco FT/IR-430 spectrometer. All the experiments were carried out by using KBr discs in which a few milligrams of the resin specimens were dispersed. Otherwise, the organic resins were analyzed by using free-standing thin films. Dynamic mechanical analyses (DMA) were performed using a Triton TTDMA with a single cantilever holder at a heating rate of 2 °C/min and a frequency of 1 Hz. The samples for the DMA experiment were prepared in a rectangular-shaped Teflon mold, and the sample dimension was 50 × 10 × 3 mm^3^.

### 3.2. Synthesis Procedures

#### 3.2.1. Reaction of Melamine with *N*,*N*-diglycidyl-4-glycidyloxyaniline: Resins (I-a) and (I-b)

The resin (I-a) was prepared by mixing 0.25 g of melamine (1.98 mmol) with 1.50 g of *N*,*N*-diglycidyl-4-glycidyloxyaniline (5.41 mmol) at 60 °C for 10 min. Afterwards, 50 mg of 4-pyrrolidinopyridine (0.34 mmol) were added, and the reaction mixture was kept at 60 °C for a further 20 min. Finally, in order to complete the crosslinking process, the temperature was increased to 80 °C and kept for 3 h.

The resin (I-b) was prepared by the same procedure described above except for the stoichiometric ratio of the reagents employed: 0.75 g of melamine (5.95 mmol) and 1.50 g of *N*,*N*-diglycidyl-4-glycidyloxyaniline (5.41 mmol).

#### 3.2.2. Reaction of Melamine with *N*,*N*-diglycidyl-4-glycidyloxyaniline and APTES: Resins (II-a) and (II-b)

The resin (II-a) was prepared by mixing 0.25 g of melamine (1.98 mmol) with 0.25 g of *N*,*N*-diglycidyl-4-glycidyloxyaniline (0.90 mmol) at room temperature for 1 h. Afterwards, 1.50 g of (3-aminopropyl)trimethoxysilane (8.37 mmol) and 20 μL of an aqueous solution of NaOH ~5 M were added, in this order, and the temperature was increased up to 60 °C for 20 min. Finally, in order to complete the crosslinking process, the temperature was increased up to 80 °C and kept for 3 h.

The resin (II-b) was prepared by the same procedure described above, except for the stoichiometric ratio of the reagents employed: 0.50 g of melamine (3.96 mmol), 1.50 g of *N*,*N*-diglycidyl-4-glycidyloxyaniline (5.41 mmol) and 0.50 g of (3-aminopropyl)trimethoxysilane (2.79 mmol).

## 4. Conclusions

Through an innovative, easy and cost-effective synthetic approach, thermosetting epoxy melamine resins were prepared. In particular, two different types of resins were obtained. Resin I was synthesized using melamine and a glycidyl derivative in the presence of a catalyst, while resin II was prepared adding also a silane derivative.

This innovative procedure presents many advantages with respect to those described in the literature for similar systems:

(i) purification steps are avoided;
(i) the procedure is solvent-free;
(iii) the crosslinking reaction is made in mild thermal conditions;
(iv) the reaction does not need a thermal control, because no uncontrolled exothermic events occur.

All the obtained resins possess interesting technological properties, such as a good thermal stability and remarkable mechanical properties. In particular, resin I was thermally stable up to about 240 °C and exhibited remarkable stiffness with storage modulus values up to 830 MPa at room temperature. At variance, resin II shows lower storage moduli, probably due to the presence of silane groups, which contribute to the enhancing of the flexibility of these materials.

Moreover, from an applicative point of view, it is worth pointing out that the proposed synthesis allows the obtaining of a workable viscous paste, with a pot life higher than 30 min, just by mixing reagents and catalyst at 60 °C for about 10 min. This paste can be easily shaped, and it is able to adhere to several kinds of surfaces.

Therefore, due to their interesting thermal and mechanical properties, the easy and solvent-free formulation and the good viscosity and workability of the pre-polymer mixtures, these materials can be considered as promising candidates for commercial applications.

## Figures and Tables

**Table 1:** Thermal properties of the epoxy resins.

Resin	Temperature at 5% weight loss (°C)	Weight loss ending temperature (°C)	Residual at 800 °C (weight%)
I-a	240	740	0
I-b	247	720	0
II-a	105	745	6
II-b	236	790	22

**Table 2:** Exothermic peak temperature (*T*_p_) and the heat of the reaction for the reaction mixtures examined.

Resin	*T*_p_ (°C)	Δ*H* (J/g)
I-a	99	504
I-b	95	440
II-a	107	241
II-b	114	287

**Table 3:** Characteristic E′ values at room temperature and 150 °C, E″ peak and the glass transition temperature (*T*_g_) values for epoxy melamine resins.

Resin	E′ _(25 °C)_ (MPa)	E′ _(150 °C)_ (MPa)	E″_peak_ (°C)	*T*_g_ (°C)
I-a	830	145	94	110
I-b	737	138	62	79
II-a	137	67	110	124
II-b	672	158	120	150
